# Microbe to Microbiome: A Paradigm Shift in the Application of Microorganisms for Sustainable Agriculture

**DOI:** 10.3389/fmicb.2020.622926

**Published:** 2020-12-21

**Authors:** Prasun Ray, Venkatachalam Lakshmanan, Jessy L. Labbé, Kelly D. Craven

**Affiliations:** ^1^Noble Research Institute, LLC, Ardmore, OK, United States; ^2^Biosciences Division, Oak Ridge National Laboratory, Oak Ridge, TN, United States

**Keywords:** cover crop, microbial consortia, mycorrhiza, rhizobacteria, rhizosphere

## Abstract

Light, water and healthy soil are three essential natural resources required for agricultural productivity. Industrialization of agriculture has resulted in intensification of cropping practices using enormous amounts of chemical pesticides and fertilizers that damage these natural resources. Therefore, there is a need to embrace agriculture practices that do not depend on greater use of fertilizers and water to meet the growing demand of global food requirements. Plants and soil harbor millions of microorganisms, which collectively form a microbial community known as the microbiome. An effective microbiome can offer benefits to its host, including plant growth promotion, nutrient use efficiency, and control of pests and phytopathogens. Therefore, there is an immediate need to bring functional potential of plant-associated microbiome and its innovation into crop production. In addition to that, new scientific methodologies that can track the nutrient flux through the plant, its resident microbiome and surrounding soil, will offer new opportunities for the design of more efficient microbial consortia design. It is now increasingly acknowledged that the diversity of a microbial inoculum is as important as its plant growth promoting ability. Not surprisingly, outcomes from such plant and soil microbiome studies have resulted in a paradigm shift away from single, specific soil microbes to a more holistic microbiome approach for enhancing crop productivity and the restoration of soil health. Herein, we have reviewed this paradigm shift and discussed various aspects of benign microbiome-based approaches for sustainable agriculture.

## Introduction

The health of soil plays an essential role in the ability of plants to produce food, fuel, and fiber for a growing world population. To keep pace, the total area of cultivated land worldwide has increased over 500% in the last five decades with a 700% increase in fertilizer use and a several-fold increase in pesticide use (Banerjee et al., 2019). In addition to being the world’s largest agricultural producers and exporters, the EU, Brazil, United States, and China also are some of the world’s largest pesticide users – each using 827 million, 831 million, 1.2 billion, and 3.9 billion pounds of pesticides, respectively, in 2016 (Donley, 2019). However, these numbers are not sustainable from either a supply-chain or environmental perspective. Thus, because natural resources are limited and their overuse pollutes the environment, the continued use of fertilizers and water to meet the demand of future global food requirements is not sustainable. Of relevance here is that agricultural intensification with high resource use and low crop diversity can negatively affect soil- and plant-associated microbiota (the so-called “phytobiome”) with subsequent impacts on critical ecosystem services (Matson et al., 1997).

There is growing evidence that aboveground plant diversity supports belowground microbial biodiversity, primarily through root exudation and rhizo-deposition (Bais et al., 2006; Eisenhauer et al., 2017; Morella et al., 2020). These more simple carbohydrates released into the soil primarily feed bacteria (Gunina and Kuzyakov, 2015) and are the most abundant near the root surface and diffuse along a gradient as distance from the root increases (Gao et al., 2011). The microbial composition is more abundant and complex in the rhizosphere, the narrow zone surrounding plant roots, with up to 10^9^ cells per gram in typical rhizospheric soil, comprising up to 10^6^ taxa (Lakshmanan et al., 2017). The more complex carbohydrates (e.g., lignin, cellulose) are largely degraded by decomposer fungi that break down these recalcitrant compounds into forms that can be used by other microbes. This conversion is largely decoupled from conventional agricultural practices, wherein the organic matter content is often lost to the system (Craven and Ray, 2019), and the carbon flux is at least partially unregulated in this regard. Again, defining nutrient fluxes with techniques like Stable Isotope Labeling (SIP) holds great potential to define and construct resilient, functioning and beneficial microbiomes that can contribute to future holistic agriculture. Thus, applying an efficient and diverse soil microbiome backed by these new technologies can facilitate and promote sustainable agriculture and can effectively contribute to meet the triple requirements of economic, social and environmental sustainability (Ray and Craven, 2016).

Historically, microorganisms that promote plant growth and nutrient acquisition have been used largely as single strains in agriculture to offset such fertilizer inputs as nitrogen and phosphorous. However, studies of natural populations suggest that groups of microbes with distinct function niches play pivotal roles in adhering and desorbing inorganic nutrients to physical surfaces, as well as breaking down organic residues and incorporating them into the soil (Lakshmanan et al., 2014; Finkel et al., 2017; Kumar and Dubey, 2020). Conceptually, such observations support the idea of the microbiome as a second genome or an extended genome of the plant (Vandenkoornhuyse et al., 2015). It is now evident that improving plant performance in a sustainable manner is beyond the binary interaction between a specific microbe or a consortium of beneficial microbes and a targeted host plant. This is a much more complex set of interactions than previously thought that requires modeling for improving predictable outcomes. In this review, we will highlight the current state of the art for the incorporation of specific plant growth-promoting microorganisms and discuss the principles and management practices for next-generation, microbiome-based approaches for sustainable agriculture.

## Application of Beneficial Microbes in Sustainable Agriculture: Past, Present and Future

Since the early 1800s, the United States Department of Agriculture has recommended the use of certain rhizobacteria to improve nitrogen fertility in leguminous crops (Schneider, 1892). Since that time, a great deal of research has been conducted on this relationship between legumes and these bacteria, now termed rhizobia, that inhabit unique structures, the nodules, that form on the roots. Rhizobia infecting these nodules are now capable of “biological nitrogen fixation,” whereby di-nitrogen is fixed into forms that can be used by the plant. Symbiotically, the bacteria trade these nitrogenous compounds to the host plant in exchange for photosynthetically derived carbon. Despite these limited applications, much remains to be learned regarding both the functional and taxonomic diversity of these symbiotic bacteria and their host plants, the role they play in the global nitrogen cycle, and ultimately, how they can best be harnessed for improving plant productivity. This is particularly true for marginal lands that are not suited for row crop production but will need to be incorporated into global food and forage production approaches moving forward. Further, such degraded lands must but regenerated with the goal of restoring soil health and productivity. Any successful endeavor in this regard must include a characterization of the soil microbiome, both taxonomically and functionally. Attempts currently are underway to fix nitrogen in such non-legumes as wheat, corn and other staple crops that produce the bulk of human food by engineering symbiotic relationships using synthetic biology approaches (Rogers and Oldroyd, 2014; Ryu et al., 2020). Such approaches would significantly impact global food supplies, and may function adequately to reduce the arable land required to meet productivity goals.

Plant growth-promoting microbes not only play critical and diverse roles in growth promotion *per se*, but also in improving various aspects of plant resilience against a wide array of biotic and abiotic stresses (Arnold et al., 2003; Sun et al., 2010; Agler et al., 2016; Azad and Kaminskyj, 2016; Singh, 2016; Oleńska et al., 2020; Rai et al., 2020). In this context, researchers globally have worked over the last several decades on plant growth-promoting microorganisms, such as root-associated mycorrhizal fungi, across a broad range of crops and encompassing a wide range of agro-climatic conditions. For perspective, Brundrett and Tedersoo (2018) recently reviewed 135 years of mycorrhizal research and reported that merely 8% of the vascular plants are non-mycorrhizal, suggesting that plant families associating with mycorrhizae have been very successful over the evolution of the plant kingdom.

Traditionally, agricultural application of beneficial microorganisms involves a few types of well-characterized microbes, such as mycorrhizal fungi or rhizobia bacteria, for which the mechanisms underlying the plant growth promotion effects are well understood. Further, most of these studies focused solely on the ability of the applied microorganisms to facilitate such specific plant growth-promoting traits as phosphate solubilization, nitrogen fixation, ACC deaminase production (Sarkar et al., 2018), siderophore production, biofilm formation, plant hormone production, biotic, and abiotic stress tolerance or resistance, among others (Weyens et al., 2009; Bhattacharyya and Jha, 2012; Singh et al., 2019). While these beneficial microorganisms can impart considerable benefits to plant growth and fitness, they are typically documented in simple, one-on-one studies, often conducted in sterile soils in greenhouse conditions. As a consequence, the effects found in such simplified conditions often fail to translate to more complex field situations (Chutia et al., 2007; Nicot et al., 2011; Parnell et al., 2016). Soil in field plots have more complex microbial environments that are presumably adapted to the local eco-environment.

In recent years, next-generation sequencing has revolutionized our understanding of microbial community composition and function, and together with improved culturing methodologies has greatly facilitated the use of biologicals in the field (Schweitzer et al., 2008; Panke-Buisse et al., 2014; Mueller and Sachs, 2015). Specifically, metagenomics-based approaches have uncovered vast, previously unrecognized populations of microbes that may have new or enhanced properties that could be used for agriculture, bioremediation, and human health. For example, comparative analyses of rhizosphere metagenomes from resistant and susceptible tomato plants enabled the identification and assembly of a flavobacterial genome that was far more abundant in the resistant plant rhizosphere microbiome than in that of the susceptible plants. Such findings certainly reveal a role for native microbiota in protecting plants from phytopathogens, and pave a way forward for the development of probiotics to ameliorate plant diseases akin to human health (Kwak et al., 2018). In another study, a 16S rRNA gene amplicon sequencing analysis of maize root microbiome led to the identification of bacteria that promote growth under low temperature conditions (Beirinckx et al., 2020). Additionally, principles of consortium design that rely on cross-talk, cross-feeding and/or substrate channeling between different microorganisms offer new opportunities for “intelligent” consortia design (Calvo et al., 2014; Vorholt et al., 2017; Paredes et al., 2018). We propose that the manipulation of the plant microbiome holds tremendous potential for agricultural improvement (Table 1). Through recent years of research, it is elucidated how microbes worked in nature before, and how decades of chemical fertilizer use have silenced their ability to improve plant fitness and soil health. Therefore, designing a microbial consortium that carefully weighs and evaluates the relationship between inoculants and the resident microbiome would substantially improve the plant growth-promoting potential and resilience of agricultural biologicals to boost plant growth. In this review, we will discuss the key considerations that would improve the likelihood of microbial products to improve crop yield, decrease disease severity and/or ameliorate abiotic stress response. Further, it is likely that such considerations would reduce the inconsistency between the performances of beneficial microbes from controlled greenhouse conditions and more natural environments.

**TABLE 1:** List of recent publications in plant and soil microbiome focusing on plant fitness and productivity.

Category	Salient findings	Reference
Plant growth promotion	Chilling temperatures critically affects growth of Maize in N. hemisphere. This study reported enrichment of *Comamonadaceae* and the *Pseudomonadaceae* in the root endosphere of maize grown under chilling conditions. Additionally two bacterial strains were identified from the root endosphere that could boost maize growth under chilling conditions.	Beirinckx et al., 2020
	A root endophytic bacteria Sphingomonas sp. Cra20 improved growth of Arabidopsis thaliana under drought stress by stimulating the growth of lateral roots and root hairs. Additionally, the relative abundance of Sphingomonas increased in the rhizosphere bacterial community in the water-deficit treatment, suggesting the role of Sphingomonas sp. Cra20 in alleviating drought induced stress.	Luo et al., 2019
	A community-based culture collection (CBC) approach was undertaken to isolate bacteria from the stalks and rhizosphere of Sugarcane. Subsequently, a synthetic community was designed by cross-referencing the CBC with the sugarcane microbiome profile that comprised of highly abundant bacterial groups from roots and stalks. The synthetic community could successfully improve the biomass of sugarcane, and was found to displace the native rhizosphere bacteria community.	Armanhi et al., 2018
	Willows (Salix spp.) were grown in gamma-irradiated petroleum-contaminated soils. Plants were inoculated with contaminated rhizosphere soil from a willow that grew well, or with contaminated bulk soil in which the plants had died. Willows inoculated with bulk soil performed better than those inoculated with rhizosphere soil. Microbiomes of different treatments were divergent at the beginning, but had converged at the end of the study, suggesting lasting effect of inoculated microbiome on plant growth, but not on the rhizosphere microbiome.	Yergeau et al., 2015
Plant defense response	Microbiome structure of Banana endosphere in the roots and shoot tips were investigated during plant growth and wilting processes. The keystone bacterial species belonging to the family *Enterobacteriaceae* family were isolated and further engineered to express ACC deaminase. Plants inoculated with engineered *Enterobacteriaceae* strains increased resistance to the Fusarium wilt disease. The findings illustrate that the keystone species in the banana microbiome plays functional role in the wilt resistance.	Liu et al., 2019
	Composts represent a sustainable way to suppress diseases and improve plant growth. Compost derived microbial communities enriched in the rhizosphere of Tomato were analyzed for antifungal activity against soil-borne fungal pathogens. Subsequently, microbial synthetic communities (SynComs) were designed with an overarching aim to improve plant fitness. SynComs were found to promote tomato growth as well as suppressed Fusarium wilt symptoms in controlled conditions.	Tsolakidou et al., 2019
	Tomato variety Hawaii 7996 is resistant to the soil-borne pathogen *Ralstonia solanacearum*, whereas the Moneymaker variety is susceptible to the pathogen. Rhizosphere microbiome analysis revealed clear differences in community profile of these two varieties. Transplantation of rhizosphere microbiota from resistant plants suppressed disease symptoms in susceptible plants. Additionally, a flavobacterium strain isolated from resistant plant rhizosphere microbiome was found to suppress R. solanacearum in susceptible plants.	Kwak et al., 2018
	A simplified synthetic bacterial community based on maize rhizosphere microbiome was designed representing most dominant phyla to study their functional attributes in maize seedlings. This synthetic community inhibited the phytopathogenic fungus *Fusarium verticillioides*, both *in planta* and *in vitro.* This study indicates how community profile information can be utilized to design beneficial microbial consortia for improving plant fitness and productivity	Niu et al., 2017
Abiotic stress response and nutrient use efficiency	Recognition of microbes by plant immune system mediated by phosphate stress was investigated. A representative synthetic bacterial community (SynCom) was designed that comprised of 35 bacteria isolated from the roots of Brassicaceae. SynCom enhanced the activity of PHR1, the master transcriptional regulator of the PSR, in Arabidopsis thaliana grown under limited phosphate. Additionally PHR1, repressed plant’s immune system in phosphorous starved regime, validating plant’s prioritization of nutritional stress over defense.	Castrillo et al., 2017
	Nitrogen-use efficiency of indica varieties of rice is superior to that of japonica varieties. Root microbiome analysis of these two varieties revealed that microbiota of indica and japonica highly distinct. Further, it was found that this distinctness was associated with a rice nitrate transporter and sensor NRT1.1B. Based on microbiome analysis, microbial synthetic communities (SynComs) were designed. It was found that indica-enriched SynCom improved rice growth in organic nitrogen conditions compared with a japonica-enriched SynCom.	Zhang et al., 2019
	Root associated microbiome of drought-sensitive pepper plant (*Capsicum annuum* L.) were analyzed focusing on role of microbes conferring plant growth under water limitation. Subsequently pepper root associated culturable bacteria were isolated and evaluated for plant growth promotion and drought tolerance abilities. The composition of the cultivable community associated to rhizosphere and root surrounding soil fractions shared a high similarity. Most of these isolates were able to promote plant growth and alleviate drought-induced stress with enhanced abilities observed in *Bacillus* and the *Rhizobacteria* strains.	Marasco et al., 2012
	Community profiling microbiome associated with superior halo-tolerant seepweed Suaeda salsa revealed that rhizospheric and endophytic bacterial community were enriched in genes responsible for salt stress acclimatization. This suggest that S. salsa preferentially recruit halotolerant taxa to confront soil salinity. Based on root endosphere core microbiota, halotolerant bacterial and fungal strains belonging to Pseudomonadales and Montagnulaceae were isolated. It was demonstrated that these core microbiome members were successfully able to improve growth and salt tolerance in the non-host rice plant.	Yuan et al., 2016

## Microbes for Plant Growth Promotion: A Reductionist Approach

**FIGURE 1:**
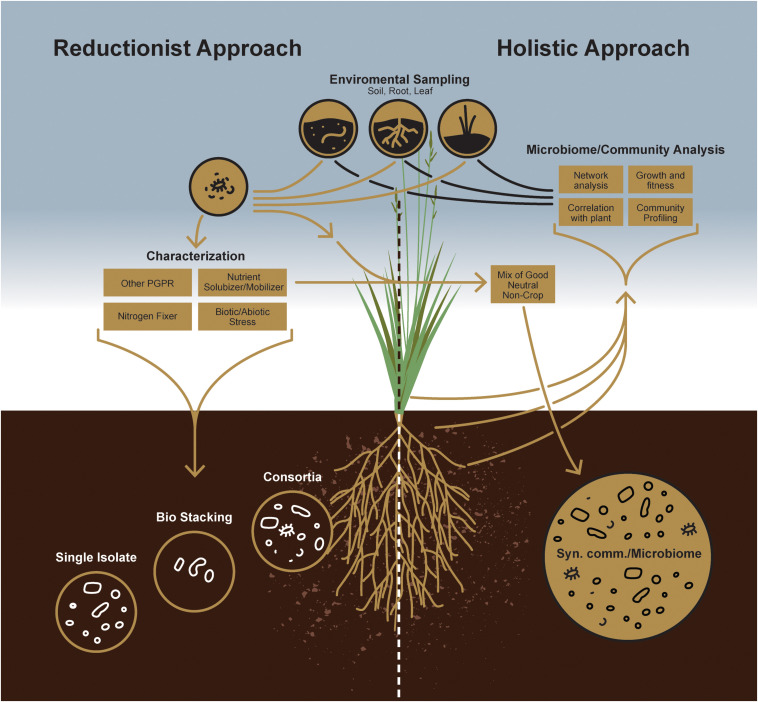
A schematic comparison between individual microorganism-based reductionist approach and microbial community-based holistic approach.

Sustainable agriculture primarily focuses on reducing the dependency of plants on chemical fertilizers and improving their ability to grow on marginal soil types. For such purposes, individual microorganisms for plant growth-promotion have largely focused on those that facilitate growth and development by enhancing acquisition of nutrient resources from the environment, including fixed nitrogen, iron and phosphate, or modulating growth by altering plant hormone levels (Figure 1) (Hayat et al., 2010). Another approach aimed at reducing yield losses to disease relies on microbes that decrease or prevent the deleterious effects of plant pathogens by several different mechanisms (Glick, 2012), i.e., by acting as a biocontrol agent. Microbe-based plant growth-promoting products, more popularly marketed as biofertilizer, has been commercially available in many countries since the 1950s (Timmusk et al., 2017). Application of such plant growth-promoting microbes in agricultural context and more specifically as inoculants has been nicely reviewed by Souza et al. (2015). However, under certain cases, the results obtained in the laboratory could not be reproduced in the field primarily due to the presence of many crop species and crop varieties, variable environmental conditions between fields, (Timmusk et al., 2017; Saad et al., 2020), occasionally due to the low quality of the inocula, and their inability to compete with the indigenous population. In that context, it is important to consider the fact that there is always greater likelihood of success by introducing mixed cultures of compatible microorganisms, rather than single, pure cultures. This is simply because each strain in the multi-strain consortium can compete effectively with the indigenous rhizosphere population and enhance plant growth with its partners. For example, sequential inoculation of nitrogen fixing bacterium *Azotobacter vinelandii*, followed by plant growth-promoting root-endophytic fungus *Serendipita indica* demonstrated better growth in rice (Dabral et al., 2020). Dual inoculation of *S. indica* and *Mycolicibacterium* strains boosted the beneficial effects in tomato (del Barrio-Duque et al., 2019) and that of arbuscular mycorrhizal fungus with plant growth-promoting bacteria *Bacillus subtilis* demonstrated better growth in wheat (Yadav et al., 2020) as compared to the singly inoculated plants. There also are numerous other reports that showed two strains used in a consortium promoted plant growth in a more effective manner (Nadeem et al., 2013; Fatnassi et al., 2015; Priyadharsini and Muthukumar, 2016). Nevertheless, to unlock the full potential of soil microbes for such nutrient cycling as nitrogen or phosphorus and providing plant protection against biotic and abiotic stress microbiomes, it is necessary to develop strategies to comprehend the functional capabilities of soil microbial communities. Irrespective of the approach, persistence is the first and foremost principle underlying the design of a successful microbial consortium for conferring plant growth promotion. This is not surprising, as the survival and activity of microbes in any soil system face a monumental task of competing with the myriad of microbes naturally adapted to that same soil. Thus, in addition to establishment of a compatible interaction with the host, a successful microbial inoculant has to subsequently compete and persist in the context of indigenous microbes as well as local abiotic conditions (Finkel et al., 2017). It has been reported that bacterial inoculations can persist in soil up to 7 weeks, but whether this inoculum also can provide plant growth benefits is not clear (Schreiter et al., 2014). While persistence or resilience of any microbial inoculum is more dependent on biotic components of a specific soil type, their persistence can be improved by inoculating crops with consortia rather than single strains (Verbruggen et al., 2012; Nemergut et al., 2013). Thus, it can arguably be stated that the diversity of a microbial inoculum, in addition to its plant growth-promoting traits, is critical for enhancing productivity and longevity (Cordero and Polz, 2014).

**FIGURE 2:**
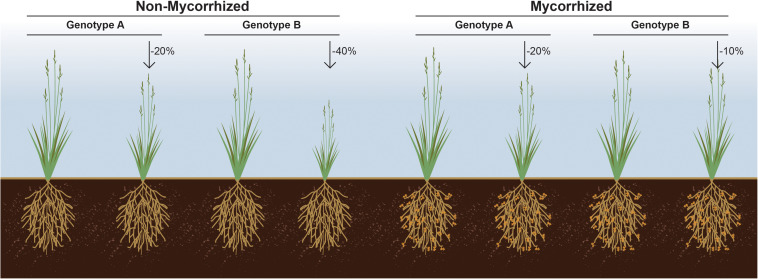
Responsiveness as the% gain in plant fitness attribute in response to symbiosis over un-colonized cohorts. This figure illustrates a hypothetical situation wherein genotype A loses less biomass (–20%) in response to soil nutrient limitation than does genotype B (–40%). However, if genotype B for its inherently associated rhizosphere microbiome responds optimally to a mycorrhizal symbiont, then it may be that it loses the least biomass (–10%) due to soil nutrient limitation, if the symbiont is present.% denotes loss in biomass due to soil nutrient limitation.

To improve the likelihood of success for such a management strategy, *a priori* knowledge of indigenous microbial populations competing with the introduced plant growth-promoting agent(s) is critical. While a reductionist approach can define the currency of individual plant-microbe interactions, the concepts of microbial community survival and functioning require, a more holistic, microbiome-based approach empowered by next-generation sequencing technology to study plant-microbe interactions at the community level (Figure 2). Indeed, this will enable researchers to design more robust, synthetic microbial consortia capable of reliably enhancing agricultural productivity.

## Microbiomes for Plant Growth Promotion: The Holistic Approach

Soil is a vastly heterogeneous growth medium, providing a wide spectrum of ecological niches for microorganisms that enable diverse strains to coexist and form complex microbial communities. When the earliest plants extended their roots into primordial soils, they encountered a habitat already teeming with bacterial and fungal life (Bulgarelli et al., 2013; Kemen, 2014). Since that early time, plants have interacted with rhizosphere microbes, evolving strategies to forge beneficial alliances with some while keeping others at bay. Such early associations certainly had consequences on plant growth and development. Therefore, a more holistic approach is needed to understand better these microbes and the roles they play in the overall health of plant and soil (Figure 1). Again, recent advances in next-generation sequencing technology and the decreasing costs associated with that technology now allow us to evaluate how microbial populations fluctuate in both space and time or to identify core microbiomes that appear conserved among host genotypes or species (Sergaki et al., 2018). Thus, although culture-independent methods have contributed tremendously to our understanding of plant-associated fungal and bacterial community structures, the study of microbiome functions remains challenging because of the inherent noise of plant-associated microbial communities. It is now well known that there are core sets of microbes that, depending on the host, are recognized as keystone taxa that consistently associate with healthy plants (Banerjee et al., 2018). Consequently, researchers working with specific plant-microbe interactions have increasingly acknowledged the mitigating impact these larger microbial communities have on individual plant-microbe outcomes for plant growth promotion or fitness. Now, plant-associated fungal and bacterial stains from various plant species are being isolated, which will provide in the near future an inestimable resource for assembling taxonomically defined microbial communities with increasing complexity. Therefore, it is now imperative to take advantage of this knowledge to design consortia of microbes to maintain a sustainable rhizosphere community, with key functional properties that include plant protection, nutrient acquisition, and alleviating biotic and abiotic stress responses. From that perspective, synthetic community (SynCom) approaches can provide functional and mechanistic insights into how plants regulate their microbiomes (Figure 1). Not surprisingly, recent culture-independent analyses thus have paved the way for developing SynComs more often (Bodenhausen et al., 2014; Armanhi et al., 2018; Carlström et al., 2019).

Mycorrhizal fungi, at least the arbuscular type, were early symbiotic partners of most land plant species, improving nutritional conditions through soil exploration and pathogen resistance of host plants (Klironomos et al., 2000). In reward for the essential physiological services, they receive ca. 20% of net photosynthetic products from plants (HoÈgberg et al., 2001). Other mycorrhizal systems may have different nutritional benefits and costs, as has been proposed for the serendipitous system (Craven and Ray, 2019). Additionally, third-party partners can modulate the outcome of the tripartite interaction, such as the case of mycorrhizal helper bacteria (Frey-Klett et al., 2007), fungal endobacteria (Bonfante and Desirò, 2017; Bonfante et al., 2019) like *Candidatus Moeniiplasma glomeromycotorum* within the spores and hyphae of Glomeromycotina (Naito et al., 2017), *Rhizobium radiobacter* within *Serendipita indica* (Guo et al., 2017), and N_2_-fixing endobacteria *Pseudomonas stutzeri* inside basidiomycetes yeast endophyte *Rhodotorula mucilaginosa* (Paul et al., 2020). Hence, it is imperative to consider the composition and functioning of these microbe–microbe interactions to understand plant–microbiome associations in a holistic manner.

## Principles and Management of Rhizosphere Microbiomes for Sustainable Agriculture

### Competence and Resilience of the Rhizosphere Microbiome: Impact of Introduced Microbes on Native Microbiomes

In 1904, the German agronomist and plant physiologist Lorenz Hiltner coined the term *rhizosphere* (Hartmann et al., 2008) to describe the area around a plant root inhabited by a unique population of microorganisms. Since then, numerous studies have been undertaken to decipher the interplay between plants and rhizosphere microorganisms, encompassing a wide variety of plant growth-promoting bacteria, fungi, insects, protozoans, viruses, etc. (Marschner, 2012; McNear, 2013). The majority of these studies have traditionally followed a simple principle for maximizing successful host infection by pre-inoculation onto the targeted crop of choice to provide a competitive advantage for a desired microbe. Conceptually, this increases the relative abundance of a given beneficial microbe in the rhizosphere, at least temporarily, to achieve the desired benefit. Such studies typically take place in a controlled, artificial condition, such as a defined growth medium in a greenhouse, where competition from a native rhizosphere community is relatively low or non-existent. As mentioned above, this approach occasionally has failed once field application is attempted or the benefits are dramatically reduced in amplitude and/or endurance.

As an example, Lekberg and Helgason (2018) conducted a literature survey of research papers published on mycorrhizal functioning spanning a 30-year period (1987–2017). The most striking finding of this survey was that less than 5% of the work scientifically manipulated mycorrhizal abundance in the field. While we are not arguing the merit of greenhouse-based studies where the number of variables can be controlled and accounted for, yield gains in field conditions will continue to be modest with such an approach. Rhizosphere competence must be evaluated in a field situation if the true power of this approach is to be realized.

Over the last few decades, mycorrhiza-based bio-fertilizers containing one or several species of fungi were developed in forestry and agriculture (Jeffries and Rhodes, 1987; Baraza et al., 2016; Igiehon and Babalola, 2017). These inoculants are generally effective in plant growth promotion under controlled lab and greenhouse conditions. However, few targeted efforts have been made to measure interactions between the introduced microbe(s) and the native mycorrhizal community, let alone the more complex rhizosphere microbiome (Svenningsen et al., 2018; Turrini et al., 2018). To optimize outcomes from these interactions, targeted research must be undertaken to understand how such mycorrhiza-based biofertilizer integrate themselves within the context of the native microbiome.

### Integration of Rhizosphere Microbiomes in Plant-Microbe-Nutrient Relationships

The soil microbial community often assists plants by weathering minerals from rock surfaces and degrading recalcitrant soil organic matter whereby soil microbes break down soluble and insoluble organic matter and convert it into inorganic, plant-available forms. Soil organic matter turnover is thus considered a net positive, as it liberates the nutrients locked up in organic matter. For this reason, conventional farming has always relied heavily on soil tillage, along with such other intensive agricultural practices as usage of inorganic fertilizers, herbicides and pesticides. However, it is already clear that such practices have negative consequences on the functional diversity of soil microbiomes. Long-term chemical fertilization has been shown to dramatically decrease the soil pH, which leads to a decrease in bacterial diversity and other changes in microbial community structure (Sun et al., 2015). This was well documented in the work of Kumar et al. (2017), who showed that long-term application of high doses of inorganic nitrogenous fertilizers severely reduces relative abundance, diversity and structure of diazotrophs, which play a key role in converting atmospheric N_2_ to plant-available ammonium.

As mentioned above, soil bacterial communities play a pivotal role in soil organic matter decomposition. In particular, soil carbon and nitrogen are critical factors for bacteria that rely on soil organic C and N decomposition to obtain energy (Chen et al., 2014; Wild et al., 2014; Tian et al., 2018). Further, different types of soil C selectively manipulate soil microbial community composition, resulting in changes in such belowground ecosystem functions as decomposition and nutrient transfer and creating feedbacks that may affect overall plant growth and productivity (Orwin et al., 2006). For example, bacteria belonging to the genera *Chloroflexi*, *Nitrospirae*, and *Planctomycetes* preferentially feed on recalcitrant organic C, whereas *Proteobacteria* and *Bacteroidetes* prefer labile organic C present in the soil (Nie et al., 2018). For this reason, amending the soil with such organic fertilizers as compost or manure contributes to higher microbial diversity and biomass compared to mineral-fertilized soils, which in turn positively impacts soil health (Schmid et al., 2018; Banerjee et al., 2019). Unfortunately, only a few agroecosystem experiments exist that compare organic and conventional management strategies over an extended period for evaluation of impact on soil health and restoration (Raupp et al., 2006; Khatoon et al., 2020). Hartmann et al. (2015) took a metagenomics approach to assess microbial diversity of soil in response to more than 20 years of continuous organic and conventional farming. Not surprisingly, they found that organic farming increased richness, decreased evenness, and shifted the structure of the soil microbiota when compared with conventionally managed soils under mineral fertilization (Hartman et al., 2018; Li et al., 2020b). There also are reports of significant alterations in the microbial community composition of both summer maize and winter wheat in response to increased nitrogen fertilization dose (Wang et al., 2018; Li et al., 2020a). Clearly, a better understanding of the interactions between the soil microbiome and conventional agricultural practices is crucial for the development of sustainable management of soil fertility and crop production.

### Managing the Rhizosphere Microbiome to Induce Disease Suppression in Soil

Disease suppressive soils were originally defined by Baker and Cook (1974) as “soils in which the pathogen does not establish or persist, establishes but causes little or no damage, or establishes and causes disease for a while but thereafter the disease is less important, although the pathogen may persist in the soil.” Disease suppressive soils are the best examples of microbiome-mediated protection of plants against root infections by soil-borne pathogens. Such disease-suppressive soils have been described for various soil-borne pathogens, including fungi, bacteria, oomycetes, and nematodes (Mazzola, 2007; Kwak et al., 2018). To date, several microbial genera have been proposed as key players in disease suppressiveness of soils, but the complexity of the microbiome, as well as the underlying mechanisms and microbial traits, remain elusive for most disease suppressive soils (Toyota and Shirai, 2018).

Recently, Carrión et al. (2019) showed that upon pathogen invasion, members of the *Chitinophagaceae* and *Flavobacteriaceae* became enriched within the plant endosphere. They proposed that this bacterial population shift led to the induction of enzymatic activities associated with fungal cell-wall degradation, as well as secondary metabolite biosynthesis, all aimed at accelerating and augmenting the plant defense response(s). Although the disease suppressive abilities of certain soils can be at least partially attributed to their physico-chemical properties, the capacity of a soil to suppress disease progression is more often attributed to agri-management practices and crop rotation (Weller et al., 2002). In classic studies by Gerlagh (1968) and Shipton et al. (1973), the authors have shown soil to become disease suppressive after mono-culturing wheat over time. More recently, a comparative metatranscriptome analysis of wheat rhizosphere microbiome grown in fields suppressive and non-suppressive to the plant pathogen *R. solani* AG8 clearly revealed distinct dominant taxa in these two soil types. Additionally, suppressive samples showed greater expression of polyketide cyclase, terpenoid biosynthesis, and cold shock proteins (Hayden et al., 2018). While development of probiotics for the human gut microbiome has already been an established field of research, the use of probiotics that comprises naturally occurring bacterial antagonists and competitors that suppress pathogens has recently emerged as a promising strategy for disease suppression in soil. A study on application of probiotic consortia that comprised predefined *Pseudomonas* species reported suppression of the bacterial plant pathogen *Ralstonia solanacearum* in the tomato rhizosphere microbiome (Hu et al., 2016). In another study, amendment of *Metarhizium*, an insect-pathogenic fungus that is commonly employed as biological control agents against crop pests, in the rhizosphere of common bean (*Phaseolus vulgaris*) significantly increased the relative abundance of plant growth promoting such taxa as *Bradyrhizobium*, *Flavobacterium*, *Chaetomium*, and *Trichoderma* while suppressing the root rot disease symptoms *Fusarium solani* (Barelli et al., 2020). Soil suppressive properties are mostly derived from the biological functions of soils. Therefore, elucidation of microbial functions in suppressive soils by a next-generation sequencing approach will facilitate the development of effective, consistent and durable disease management tools.

### Impact of Agriculture Management Practices on the Soil Microbiome

One important context for plant-microbe interactions is soil structure, as it can vary greatly depending on land-use history, plant species composition and successional stage (Erktan et al., 2016). Besides playing pivotal roles in soil organic matter decomposition, carbon cycling, nutrient mobilization, etc., saprotrophic fungi also are involved in creating soil structure through the secretion of extracellular compounds and physical binding of soil via hyphal networks (Bergmann et al., 2016). Interestingly, studies on the impact of tillage on the soil fungal communities have shown mixed results. Reports in no-till systems have varied from increased ratios of fungal to bacterial biomass (Acosta-Martínez et al., 2010) to decreased ratios (Mbuthia et al., 2015), as well as no change at all (Mathew et al., 2012). More recent studies have shown that soil fungal communities are negatively impacted by tillage, as they typically would be responsible for degrading crop residue left on the surface with no-till (Yin et al., 2017). More specifically, soil bacterial communities were primarily found to be structured by tillage, whereas soil fungal communities responded mainly to management type with additional effects by tillage (Hartman et al., 2018). Additionally, it is acknowledged that organically managed systems increased taxonomic and phylogenetic richness, diversity and heterogeneity of the soil microbiota when compared with conventional farming systems (Lupatini et al., 2017). In a simple definition, organic farming system consists of low-input agro-ecosystem farms in which plant productivity and ecosystem functionality are based on the natural availability of plant nutrients (Lammerts van Bueren et al., 2002). A study aimed at comparing the soil microbiome in conventional and organic farming systems in central Europe revealed no major differences among the main phyla of bacteria between the two farming styles (Armalytë et al., 2019), whereas another study that investigated the effects of 12 years of organic farming on soil microbiomes in northern China reported shifting of the community composition of dominant phyla and significant alterations of functional groups associated with ammonia oxidation, denitrification and phosphorus recycling when compared to conventional farming systems (Ding et al., 2019).

In addition to tillage, crop rotation also plays a pivotal role in increasing belowground microbial diversity compared to intensive mono-cropping practices. Although the United States Department of Agriculture has advocated [via the Conservation Reserve Program (CRP)] crop rotation to improve eroded land as early as 1985 (Allen and Vandever, 2005), its benefit on soil health has only been recognized recently. Several studies reported increases in such soil quality parameters as organic matter content, microbial biomass and respiration under crop rotation management when compared with a mono-cropping system (Campbell et al., 1991; Luce et al., 2013). A meta-analysis of 122 studies that examined crop rotation revealed similar findings, namely that adding one or more crops in rotation to a monoculture substantially increased the soil microbial biomass along with increases in total soil C and N, respectively (McDaniel et al., 2014). In another study, soil microbial communities of corn and switchgrass in mono-cropping systems when compared with mixed prairie grasses demonstrated that bacterial and fungal biomass, especially arbuscular mycorrhizal fungi, were higher in plots with mixed prairie grasses (Jesus et al., 2016). A 16S amplicon-based metagenomic analysis of an almost 20-year-old field trial in Bernburg, Germany revealed a significant effect of tillage practice and the preceding crop on prokaryotic community structures (Babin et al., 2019)

Cover crops are typically unharvested crops planted between cash crops that augment C provisioning to the soil system not only via unharvested residues, but also as root exudates that can support many rhizosphere microbes during the active growing season of the cover crop. Other benefits attributed to cover cropping include improved N fertility by incorporating legumes as a cover crop, reduced soil compaction via deep-rooted plants, and reduced erosion by keeping a plant and its root system in the field year round (Fernandez et al., 2016). Of various crop rotation management practices, those that include cover crops sustain soil quality and productivity by enhancing soil C, N and microbial biomass (Kim et al., 2020), making them a cornerstone for sustainable agroecosystems. Nonetheless, very few studies have assessed the relationship between cover crop stands and their associated belowground microbial communities. Early research in unfertilized grasslands demonstrated that fungal communities respond positively to plant-derived C inputs, suggesting that inclusion of cover crops in a rotation may promote fungal community development (Denef et al., 2009). More recently, a field study tested this hypothesis by specifically examining the impact on soil microbial communities of eight fall-sown cover crop species grown singly and in multispecies mixtures following a spring oats (*Avena sativa* L.) cropping season and found that certain cover crops selectively favored particular microbial functional groups. Arbuscular mycorrhizal fungi were more abundant beneath oat and cereal rye (*Secale cereale* L.) cover crops, while non-AM fungi were positively associated with hairy vetch (*Vicia villosa* L.) (Finney et al., 2017). Beyond positively affecting soil C and increasing the diversity of such beneficial fungi as arbuscular mycorrhiza, clover as a cover crop is often reported to suppress the relative abundance of pathogenic fungi (Benitez et al., 2016). Contrarily, in a 2-year field study, cover crops reportedly increased overall phylogenetic diversity of fungi but did not change the relative abundance of saprophytes, symbionts or pathogens, implying that cover cropping does not always appear to contribute to functional changes in the fungal community (Schmidt et al., 2019).

### Reassessment of Plant Responsiveness to Symbiosis

It is now increasingly evident that plants employ fine-tuned mechanisms to shape the structure and function of their microbiome, with different genotypes of the same plant species growing in the same soil yet associating with distinct microbial communities (Berendsen et al., 2012). This is demonstrated in the findings of Bazghaleh et al. (2015), who clearly demonstrated the importance of intraspecific host variation in the association of chickpea cultivars with AM and non-AM fungi. Therefore, specific traits of a plant that modulate its microbiome should be considered as a trait for plant breeding (Wallenstein, 2017).

Despite the obvious importance of beneficial microorganisms for plant growth and fitness, and the impact of plant genotype on shaping their microbiome composition, plant germplasm is typically screened in the absence of microbes, and the selection of best breeding lines made solely based on the interaction between plant genotype and performance under various abiotic factors. We propose that an *a priori* examination of the interaction between a plant genotype(s) and the symbiotic microbes upon which it likely depends is an important factor in the selection of plant breeding lines. It seems very likely that a subset of rejected germplasm could outperform others, but only when coupled with a beneficial microbe or microbiome (Figure 2). Arguably, current breeding and selection efforts most likely result in decoupling of the soil microbiome from plant fitness. As a result, modern varieties may have lost their ability to support diverse microbiomes and thus, fail to gain the most from these interactions (Wallenstein, 2017).

It is now acknowledged that transitioning from a highly intensive mono-cropping system to a more diversified cropping system consisting of multiple host genotypes leads to increased bacterial and fungal diversity (Calderon et al., 2016). Hence, future plant breeding efforts should incorporate plant characteristics that are related to microbiome diversity. For example, efforts focusing on manipulating plant root exudates likely play a critical role in selective recruitment of the rhizosphere microbiome (Bakker et al., 2012). In support of this notion, it has been shown that plants can select which microbial populations receive the lion’s share of root exudates, demonstrating a capacity by the host to refine its microbial composition. Hence, an unbiased screening of plant genotypes for responsiveness in the presence of a beneficial microbe or microbiome can set forth a new and potentially transformative paradigm in selecting microbes for plant growth promotion (Figure 2).

## Significance of Mycorrhizas: A Critical Component of Healthy Soil Rhizospheres

Mycorrhizae are mutualistic associations between soil fungi and plant roots that gradually evolved to be reciprocally beneficial to both partners (Brundrett, 2002). The benefits are generally assumed to involve an exchange of photosynthetically derived carbon from the host plant in exchange for soil nutrients provided by the foraging mycorrhiza. While likely true of arum-type arbuscular types of mycorrhizae, there are other types that can derive carbon from organic matter in the soil, or even “steal” it from one host plant to supply to another (Allen and Allen, 1991). A recent study has reported that in contrast to *Arum maculatum*, in which carbon is entirely derived from photo-assimilation, the green leaves of *Paris quadrifolia* contain a striking 50% carbon of fungal origin. Such partial mycoheterotrophy could thus potentially be widespread among the roughly 100,000 plant species that are known to develop a Paris-type AM, with far-reaching implications for our understanding of C trading in plant-microbe communities (Giesemann et al., 2019). Exactly what the mycorrhiza gains from this interaction is still under debate, but benefits may involve a safe haven from the open, more competitive soil space and a second, more reliable carbon source (Sapp, 2004).

Mycorrhizae not only shape plant communities, but they also affect the functional diversity of their cohabitants in the rhizospheric microbiome. The mycelium of mycorrhizal fungi transports plant-derived carbon into the soil in the form of sugars, amino acids and polyols to help sustain the microbiome (Tarkka et al., 2018). More recent studies focusing on soil microbial ecology revealed that mycorrhizal fungi mediate many diverse interactions within the soil “mycorrhizosphere,” including pathogens and mutualists that fix atmospheric nitrogen, take up phosphorus, produce vitamins, and/or protect against antagonists (Buée et al., 2009; Tedersoo et al., 2020). The “ectomycorrhizosphere,” which forms a very specific interface between soil and many trees, hosts a large and diverse community of microorganisms that likely play roles in mineral weathering and solubilization processes (Uroz et al., 2007). This carbon-rich mycorrhizosphere also supports large communities of root-associated microorganisms that further accelerate weathering of minerals by excreting organic acids, phenolic compounds, protons, and siderophores (Drever and Vance, 1994; Illmer et al., 1995).

Similarly, the extraradical hyphae of arbuscular mycorrhiza provide a direct pathway for the translocation of photosynthetically derived carbon to the soil, leading to the development of nutrient-rich niches for other soil microorganisms, particularly bacteria. A quantitative real-time PCR method detected significantly higher 16S rDNA abundance in both the bulk and the rhizosphere soils of zucchini (*Cucurbita pepo* L.) inoculated with *Acaulospora laevis* and *Glomus mosseae* (Qin et al., 2014). Additionally, arbuscular mycorrhizae have been reported to increase the relative abundance of *Firmicutes*, *Streptomycetes*, *Comamonadaceae*, and *Oxalobacteraceae* inhabiting the mycorrhizosphere (Offre et al., 2007; Nuccio et al., 2013). While there is clear evidence that microbial communities in the rhizosphere function cohesively with their mycorrhizal partner in nutrient mobilization from soil minerals, nitrogen cycling and protection of plants against root pathogens, such bidirectional synergy is not always universal. There are reports that indicate suppressive effects of bacterial communities on mycorrhizal functioning and vice versa. While one study reported (Svenningsen et al., 2018) that soil with a higher abundance of *Acidobacteria* suppresses the normal functioning of extra-radical mycelium in arbuscular mycorrhizae, another study found that *Glomus intraradices* and *Glomus mosseae* suppressed most of the associated soil microbial community (Welc et al., 2010).

## A Novel Type of Endophytic Symbiont: The *Serendipitaceae*

A diverse group of fungi in the Basidiomycota, the *Serendipitaceae* (formerly Sebacinales Group B) (Oberwinkler et al., 2014) encompasses endophytes and lineages that repeatedly evolved ericoid, orchid and ectomycorrhizal types. Accordingly, in many natural ecosystems these fungi form mycorrhizal symbioses with an astounding variety of host plants – every mycorrhizal type, in fact, except for arbuscular. Previous research performed in our lab with a strain of this group, *Serendipita vermifera*, demonstrated plant growth-promoting properties in a variety of plants (Ghimire and Craven, 2011; Ray et al., 2015; Ray and Craven, 2016; Ray et al., 2020). Unfortunately, the agronomic utility of these fungi is hampered by the paucity of strains available, the large majority isolated from Australian orchids. We have begun to address this constraint by isolating the first North American strain of *Serendipita*, named *Serendipita vermifera* subsp. *bescii* NFPB0129, from the roots of a switchgrass plant in Ardmore, Oklahoma (Craven and Ray, 2017; Ray et al., 2018).

As mentioned above, soil organic matter has a tremendous influence on the biological, chemical, and physical properties of soils, making it a vital component of healthy agricultural systems. Whether a natural soil or an agricultural one, the release of the nutrients locked within SOM requires decomposers, primarily insects, fungi, and bacteria, to secrete organic acids and enzymes that can loosen and break down the cellulose and the recalcitrant lignin into nutritive forms that can be used by other microbes and plants. Unlike arbuscular mycorrhizae, which exchange inorganic, mineralized nutrients mined from the soil for carbon derived from host photosynthesis, members of the *Serendipitaceae* studied thus far have a complete arsenal of carbohydrate-active enzymes (CAZymes), representing approximately 4% of the entire gene set and rivaling the more well-studied saprophytic white and brown wood rotters, and much more than other symbiotic fungi. Additionally, genome analysis of *S. bescii* and *S. vermifera* suggests that *Serendipitaceae* fungi have the metabolic capacity to assimilate N from organic forms of N-containing compounds (Ray et al., 2019). We hypothesize that this carbohydrate-degrading enzyme complement endows these *Serendipitaceae* fungi with saprotrophic abilities (Craven and Ray, 2019). Unlike free-living decomposers that maintain a solitary lifestyle, seeking only dead or dying plant tissues as their source of subsistence, *Serendipitaceae* fungi seem to maintain a largely symbiotic lifestyle with the roots of living host plants. It currently is unclear whether there is expression of CAZymes while strains of *Serendipita* are in symbiosis with host plants, and if so, whether there is spatial or temporal separation from more mutualistic traits. Still, the capacity of some strains to form mycorrhizal relationships with orchids, where the seeds require carbon from the fungus for germination and often well into the plant’s lifespan, suggests that these *Serendipitaceae* symbionts may be less of a carbon cost to their host plant. Presumably, this saved carbon could potentially be used for other symbiotic relationships or developmental processes. In any case, these intriguing fungi and their seemingly unlimited host range provide a novel symbiosis that could be used in a broad variety of cropping systems.

## Conclusion

Soil-dwelling microorganisms are critical components of soil health, itself a determinant of plant productivity and stress tolerance. Deploying microbes to improve agriculture productivity is an extremely attractive approach that is non-transgenic and can be viewed collectively as the extended plant genome. Because these same microbes can contribute to restoring soil health and productivity, they have a bright future in low-input, sustainable agriculture that extends beyond more classically defined plant-microbe symbioses.

## Author Contributions

PR and KC conceived and planned the overall idea of the review manuscript. PR, VL, JL, and KC wrote the manuscript. All authors contributed to the article and approved the submitted version.

## Conflict of Interest

The authors declare that the research was conducted in the absence of any commercial or financial relationships that could be construed as a potential conflict of interest.
